# Oral Prion Neuroinvasion Occurs Independently of PrP^C^ Expression in the Gut Epithelium

**DOI:** 10.1128/JVI.01010-18

**Published:** 2018-09-12

**Authors:** Alison Marshall, Barry M. Bradford, Alan R. Clarke, Jean C. Manson, Neil A. Mabbott

**Affiliations:** a The Roslin Institute and Royal (Dick) School of Veterinary Sciences, University of Edinburgh, Easter Bush, Midlothian, United Kingdom; b School of Biosciences, Cardiff University, Cardiff, United Kingdom; c Centre for Clinical Brain Sciences, University of Edinburgh, Edinburgh, United Kingdom; Rocky Mountain Laboratories

**Keywords:** Peyer's patches, PrP, gut epithelium, intestine, prions, transmissible spongiform encephalopathies

## Abstract

The early replication of certain prion strains within Peyer's patches in the small intestine is essential for the efficient spread of disease to the brain after oral exposure. Our data show that orally acquired prions utilize specialized gut epithelial cells known as M cells to enter Peyer's patches. M cells express the cellular isoform of the prion protein, PrP^C^, and this may be exploited by some pathogens as an uptake receptor to enter Peyer's patches. This suggested that PrP^C^ might also mediate the uptake and transfer of prions across the gut epithelium into Peyer's patches in order to establish infection. Furthermore, the expression level of PrP^C^ in the gut epithelium could influence the uptake of prions from the lumen of the small intestine. To test this hypothesis, transgenic mice were created in which deficiency in PrP^C^ was specifically restricted to epithelial cells throughout the lining of the small intestine. Our data clearly show that efficient prion neuroinvasion after oral exposure occurred independently of PrP^C^ expression in small intestinal epithelial cells. The specific absence of PrP^C^ in the gut epithelium did not influence the early replication of prions in Peyer's patches or disease susceptibility. Acute mucosal inflammation can enhance PrP^C^ expression in the intestine, implying the potential to enhance oral prion disease pathogenesis and susceptibility. However, our data suggest that the magnitude of PrP^C^ expression in the epithelium lining the small intestine is unlikely to be an important factor which influences the risk of oral prion disease susceptibility.

**IMPORTANCE** The accumulation of orally acquired prions within Peyer's patches in the small intestine is essential for the efficient spread of disease to the brain. Little is known of how the prions initially establish infection within Peyer's patches. Some gastrointestinal pathogens utilize molecules, such as the cellular prion protein PrP^C^, expressed on gut epithelial cells to enter Peyer's patches. Acute mucosal inflammation can enhance PrP^C^ expression in the intestine, implying the potential to enhance oral prion disease susceptibility. We used transgenic mice to determine whether the uptake of prions into Peyer's patches was dependent upon PrP^C^ expression in the gut epithelium. We show that orally acquired prions can establish infection in Peyer's patches independently of PrP^C^ expression in gut epithelial cells. Our data suggest that the magnitude of PrP^C^ expression in the epithelium lining the small intestine is unlikely to be an important factor which influences oral prion disease susceptibility.

## Abstract (precis)

The accumulation of orally acquired prions within Peyer's patches in the small intestine is essential for the efficient spread of disease to the brain. Little is known of how the prions initially establish infection within Peyer's patches. Some gastrointestinal pathogens utilize molecules, such as the cellular prion protein PrP^C^, expressed on gut epithelial cells to enter Peyer's patches. Acute mucosal inflammation can enhance PrP^C^ expression in the intestine, implying the potential to enhance oral prion disease susceptibility. We used transgenic mice to determine whether the uptake of prions into Peyer's patches was dependent upon PrP^C^ expression in the gut epithelium. We show that orally acquired prions can establish infection in Peyer's patches independently of PrP^C^ expression in gut epithelial cells. Our data suggest that the magnitude of PrP^C^ expression in the epithelium lining the small intestine is unlikely to be an important factor which influences oral prion disease susceptibility.

## INTRODUCTION

Prions cause chronic neurodegenerative diseases that affect humans and some domesticated and free-ranging animal species for which there are no treatments. Bovine spongiform encephalopathy (BSE) prions also have zoonotic potential (1), exerting high societal and economic costs. The precise nature of the infectious prion is uncertain, but an abnormal, relatively proteinase-resistant isoform (PrP^Sc^) of the host cellular prion protein (PrP^C^) copurifies with prion infectivity in diseased tissues (2), and host cells must express cellular PrP^C^ to sustain prion infection (3).

Many natural prion diseases are acquired by oral consumption of contaminated food or pasture. The gut-associated lymphoid tissues (GALT) within the lining of the intestine, such as the tonsils, Peyer's patches, appendix, and colonic and cecal patches, together with the mesenteric lymph nodes (MLN), help to provide protection against intestinal pathogens. However, orally acquired prions exploit the GALT to achieve host infection (4–8). The early replication of prions within Peyer's patches in the small intestine is essential for their efficient spread from the gut to the brain (termed neuroinvasion), as oral prion disease susceptibility is blocked in their absence (5, 9–11).

Orally acquired prions utilize an elegant cellular relay in the GALT in order to establish host infection. After ingestion, the prions are first transported across the follicle-associated epithelium (FAE), which covers the lumenal surface of Peyer's patches by M cells (12–16). The prions are then acquired by mononuclear phagocytes within the GALT, which they appear to use as “Trojan horses” to shuttle them toward the follicular dendritic cells (FDC) in the B cell follicles (17–19). The subsequent replication of the prions on FDC is essential for efficient neuroinvasion from the intestine (4, 5, 17, 20). The prions then infect nearby enteric nerves before spreading along fibers of the sympathetic and parasympathetic nervous systems to the brain, where they ultimately cause neurodegeneration and death (17, 21).

M cells are specialized, highly phagocytic, intestinal epithelial cells that facilitate the uptake and transepithelial transfer of particulate antigens and microorganisms into the GALT from the gut lumen (22). The transcytosis of particulate antigens by M cells is an important initial step in the induction of efficient mucosal immune responses against certain pathogenic bacteria (23, 24) and the commensal bacterial flora (25). However, some orally acquired bacterial (26–28) and viral (29, 30) pathogens utilize M cells to achieve host infection. Prions also exploit M cells in order to enter Peyer's patches and establish host infection (13, 16). Furthermore, the density of M cells in the gut epithelium directly limits or enhances disease susceptibility. In the specific absence of M cells, the accumulation of prions in Peyer's patches and subsequent spread of the disease to the brain are blocked (13, 16). In contrast, increased M cell density at the time of oral exposure enhances prion disease susceptibility approximately 10-fold by increasing the uptake of prions from the gut lumen (16).

M cells are considered to express a variety of “immunosurveillance” receptors on their apical surfaces, which enable them to acquire certain pathogens and antigens. For example, glycoprotein 2 (GP2) can act as a receptor for FimH^+^ bacteria such as Escherichia coli and Salmonella enterica serovar Typhimurium (23). Uromodulin (also known as Tamm-Horsfall protein) may similarly mediate the uptake of certain strains of Lactobacillus acidophilus (31). Some pathogenic microorganisms appear to use receptors on M cells to aid host infection. The complement C5a receptor is expressed on the apical surface of M cells and aids the uptake of Yersinia enterocolitica to establish infection (32). Interactions between the type A1 botulinum neurotoxin complex and GP2 on the M cell surface have also been shown to mediate the intestinal translocation of the toxin in order to exert its toxic effects (33). M cells express the cellular isoform of the prion protein, PrP^C^, on their apical surfaces (26, 34). Data suggest that the pathogenic Gram-negative bacterium Brucella abortus utilizes PrP^C^ on the M cell surface as an uptake receptor to enter Peyer's patches (26).

Whether the uptake and transcytosis of prions across the gut epithelium into Peyer's patches in order to establish infection predominantly occurs via constitutive sampling of the lumenal contents or via binding to specific receptors such as PrP^C^ is not known. Treatments that impede the early accumulation of prions within the GALT can impede their spread to the brain and reduce disease susceptibility (4, 13, 16, 18). Thus, the identification of the molecular factors that facilitate the uptake of prions into the GALT will help the design of novel intervention targets and enhance our understanding of the factors that influence the risk of infection. Therefore, in the current study, transgenic mice were created in which *Prnp* expression (encoding PrP^C^) was specifically ablated in epithelial cells throughout the lining of the small intestine. These mice were then used to determine whether the absence of PrP^C^ expression in the epithelium lining the small intestine influences oral prion disease susceptibility and the early replication of prions in the GALT.

## RESULTS

### Conditional ablation of *Prnp* throughout the small intestinal epithelium.

**FIG 1:**
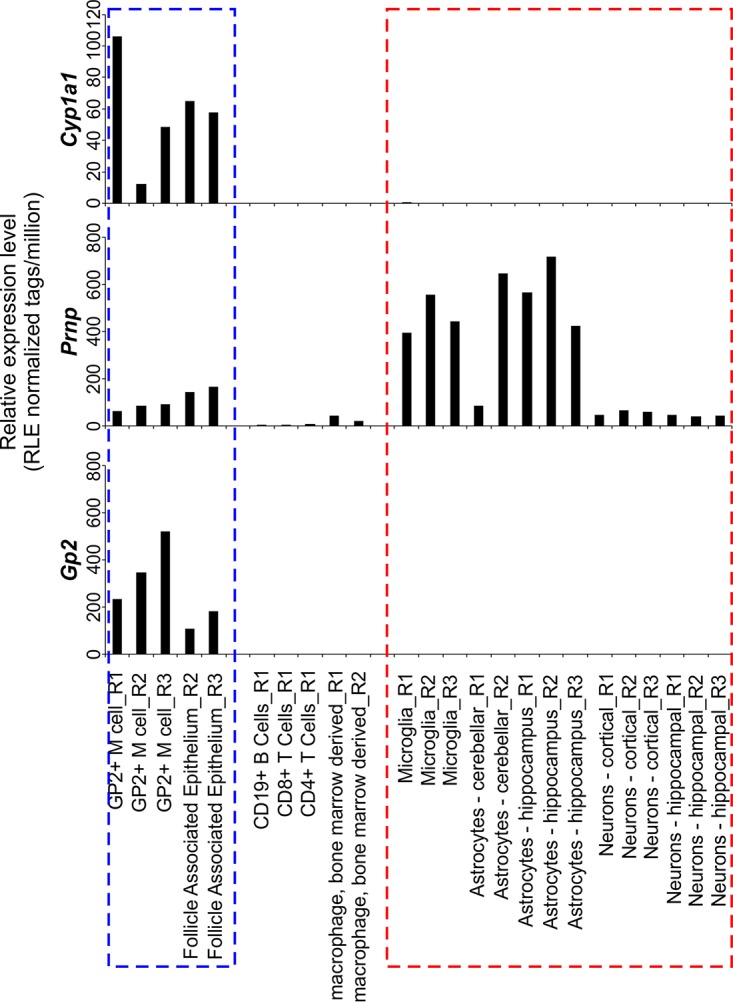
*Cyp1a1* is expressed in the follicle-associated epithelium and in M cells in the small intestine. Shown is a comparison of *Cyp1a1*, *Prnp*, and *Gp2* mRNA expression levels in individual cell populations in deep cap analysis of gene expression (CAGE) sequence data from the FANTOM5 project of the FANTOM consortium (38). Each bar shows the relative expression level of each gene per million reads in each sample [RLE normalized tags/million]. The blue-hatched box highlights the small intestine-derived glycoprotein 2-expressing (GP2^+^) M cell and the follicle-associated epithelium data sets. The red-hatched box highlights the brain-derived data sets.

The expression of Cre recombinase under the control of the rat *Cyp1a1* promoter element in *Cyp1a1*-Cre mice has been used in a series of studies to inducibly ablate the expression of *LoxP* site-flanked target genes in small intestinal progenitor cells and intestinal epithelial cells (IEC) following β-naphthoflavone (βNF) treatment (35–37). The FANTOM5 project of the FANTOM consortium (38) has collated a large collection of cap analysis of gene expression (CAGE) data from multiple mouse tissues and cells (http://fantom.gsc.riken.jp/zenbu). We used this publicly available data resource to compare the expression levels of *Cyp1a1*, *Gp2*, and *Prnp* in multiple data sets derived from mouse FAE, M cells, lymphocytes, leukocytes, and brain-derived cells. This analysis confirmed that *Cyp1a1* and *Prnp* were expressed highly in the FAE and in GP2^+^ M cells (Fig. 1). However, *Cyp1a1* expression was absent in B cells, T cells, and macrophages as well as in brain-derived microglia, astrocytes, and neurons (Fig. 1).

Here, *Cyp1a1*-Cre mice were crossed with *Prnp*^F/F^ mice, which carry a “floxed” *Prnp* gene (39), to enable the inducible ablation of *Prnp* specifically in IEC. Since the reliable detection of PrP^C^ in the gut epithelium by immunohistochemistry (IHC) is technically challenging, these mice were additionally crossed with ROSA26^F/F^ reporter mice (40) to enable the cellular specificity of the Cre-mediated gene ablation to be readily assessed by histological assessment of β-galactosidase (*LacZ*) expression. The resultant progeny *Cyp1a1*-Cre ROSA26^F/F^ *Prnp*^F/F^ mice were termed *Prnp*^ΔIEC^ mice here.

**FIG 2:**
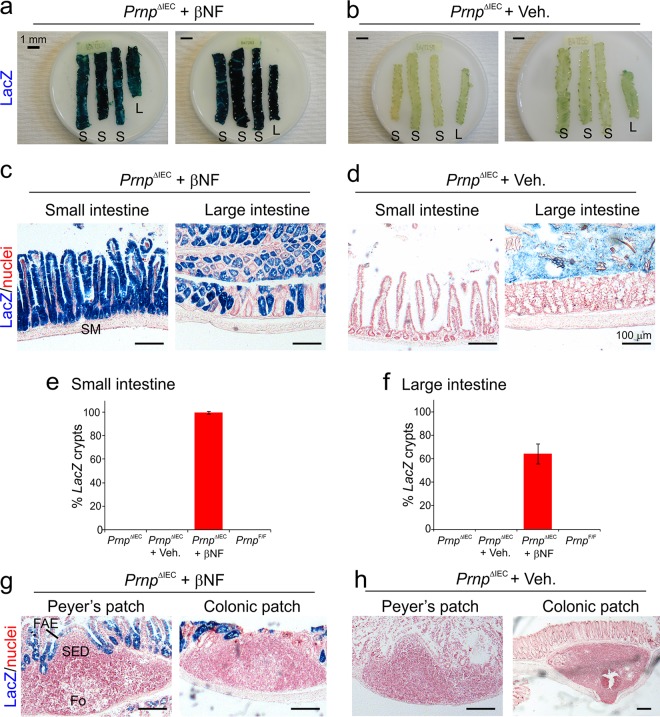
Cre-mediated gene recombination is restricted to IEC in the small intestines of βNF-treated *Prnp*^ΔIEC^ mice. Female *Prnp*^ΔIEC^ mice were treated with β-naphthoflavone (βNF) for 5 days to specifically ablate *Prnp* expression in intestinal epithelial cells, and tissues were analyzed 14 days later. *Prnp*^ΔIEC^ mice treated with the vehicle alone (Veh.) were used as controls. (a and b) Whole-mount histological analysis of *LacZ* expression (blue) in the intestines of βNF-treated *Prnp*^ΔIEC^ mice (a) or vehicle-treated *Prnp*^ΔIEC^ control mice (b). S, small intestine; L, large intestine. (c and d) Histological analysis of *LacZ* expression (blue) in IEC and crypts in the intestines of βNF-treated *Prnp*^ΔIEC^ mice (c) or vehicle-treated *Prnp*^ΔIEC^ control mice (d). Sections were counterstained with nuclear fast red to detect cell nuclei (red). SM, submucosa. (e and f) Comparison of the percentages of *LacZ*-expressing crypts in the small (e) and large (f) intestines of βNF-treated *Prnp*^F/F^ control mice. Untreated *Prnp*^ΔIEC^ mice, vehicle-treated *Prnp*^ΔIEC^ mice, and βNF-treated *Prnp*^F/F^ mice were used as controls. Data represent mean percentages of *LacZ*-expressing crypts/mouse (*n* = 5 mice/group; 50 to 105 crypts/mouse). (g and h) Histological analysis of *LacZ* expression (blue) in Peyer's patches and colonic patches of βNF-treated *Prnp*^ΔIEC^ mice (g) or vehicle-treated *Prnp*^ΔIEC^ control mice (h). SED, subepithelial dome; Fo, follicle.

Female *Prnp*^ΔIEC^ mice were treated with βNF (or the vehicle alone as a control) for 5 days to specifically ablate *Prnp* expression in IEC, and tissues were analyzed 14 days later. Whole-mount histological analysis showed *LacZ* expression indicative of efficient Cre-mediated gene recombination throughout the small and large intestines of βNF-treated *Prnp*^ΔIEC^ mice (Fig. 2a). Analysis of tissue sections showed strong *LacZ* expression in IEC and crypts throughout the small intestine (Fig. 2c). The Cre-mediated gene recombination in the small intestinal crypts of βNF-treated *Prnp*^ΔIEC^ mice was highly efficient (99.5% ± 1.1%) (Fig. 2e). In contrast, the Cre-mediated gene recombination in colonic crypts and IEC in the large intestine was less efficient (64.1% ± 8.6%) (Fig. 2f) and presented as a mosaic pattern (Fig. 2c). No other cellular sites of Cre-mediated recombination were observed throughout the intestines of βNF-treated *Prnp*^ΔIEC^ mice. *LacZ* expression was absent within the submucosa (Fig. 2c) and also in the subepithelial dome and FDC-containing B cell follicle regions of the GALT (Fig. 2g). As anticipated, no *LacZ* expression was detected throughout the small and large intestines of vehicle-treated *Prnp*^ΔIEC^ control mice (Fig. 2b, d to f, and h). *LacZ* expression was also undetectable throughout the small and large intestines of untreated *Prnp*^ΔIEC^ control mice and βNF-treated *Prnp*^F/F^ (Cre-deficient) control mice (Fig. 2e and f). These data clearly demonstrate that Cre-mediated gene recombination is restricted to IEC in the small intestines of βNF-treated *Prnp*^ΔIEC^ mice.

### Effect of IEC-restricted *Prnp* ablation on prion accumulation in lymphoid tissues.

**FIG 3:**
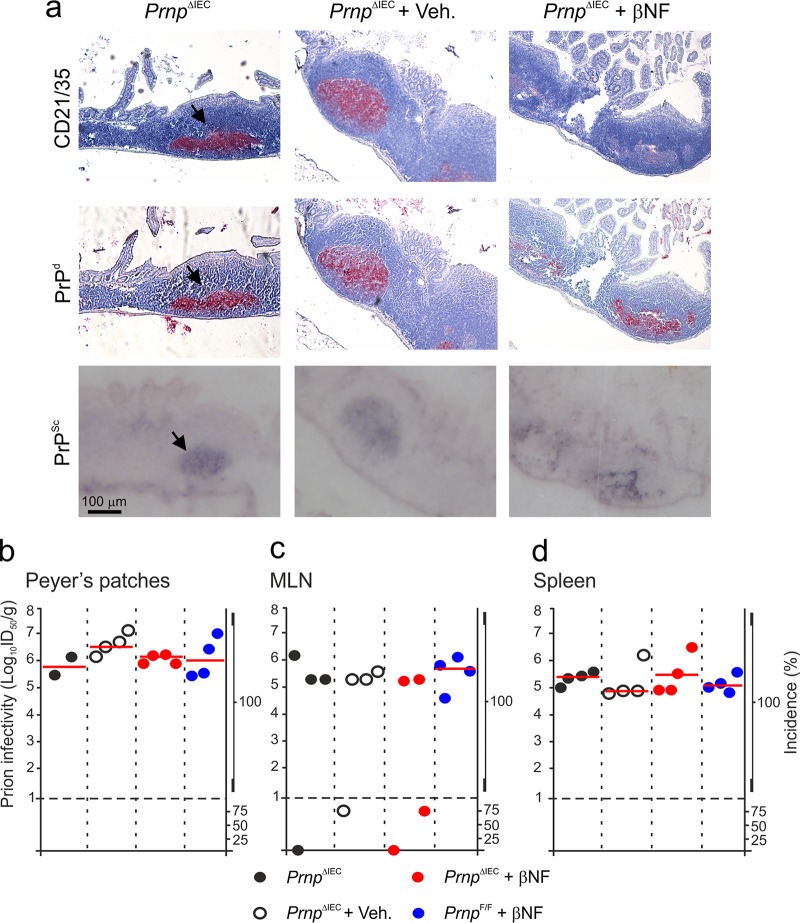
Effect of intestinal epithelial cell-restricted *Prnp* ablation on prion accumulation in lymphoid tissues. Female *Prnp*^ΔIEC^ mice were treated with βNF for 5 days to specifically ablate *Prnp* expression in intestinal epithelial cells. Untreated *Prnp*^ΔIEC^ mice and *Prnp*^ΔIEC^ mice treated with the vehicle alone (Veh.) were used as controls. Fourteen days later, the mice were orally exposed to ME7 scrapie prions, and Peyer's patches, mesenteric lymph nodes (MLN), and spleens were collected at 70 days postinfection. (a) Immunohistochemical analysis reveals high levels of disease-specific PrP (PrP^d^) (middle row, red, arrows) detected in association with FDC (CD21/35^+^ cells) (top row, red) in Peyer's patches from mice from each group. Sections were counterstained with hematoxylin to detect cell nuclei (blue). Analysis of adjacent sections by paraffin-embedded tissue immunoblotting confirmed the presence of prion-specific PK-resistant PrP^Sc^ (blue/black). Data are representative of results for tissues from 6 mice/group. (b to d) Prion infectivity levels were assayed in Peyer's patches (b), MLN (c), and spleens (d) from mice from each group collected at 70 days postinfection. Prion infectivity titers (log_10_ i.c. ID_50_ per gram of tissue) were determined by injection of tissue homogenates into groups of C57BL/Dk indicator mice (*n* = 4 recipient mice/tissue). Each symbol represents data derived from an individual tissue. Red line, median prion infectivity titer for groups in which all samples contained >1 log_10_ i.c. ID_50_/g tissue. Data below the broken horizontal line indicate disease incidence in the recipient mice of <100%, which were considered to contain trace levels of prion infectivity.

To determine the effects of IEC-specific PrP^C^ deficiency on oral prion disease pathogenesis, groups of female *Prnp*^ΔIEC^ mice were treated with βNF for 5 days to specifically ablate *Prnp* expression in IEC. Untreated *Prnp*^ΔIEC^ mice, vehicle-treated *Prnp*^ΔIEC^ mice, and βNF-treated *Prnp*^F/F^ (Cre-deficient) mice were used as controls. Fourteen days later, 10 mice/group were subsequently orally exposed to ME7 scrapie prions, and tissues were collected at 70 days postinfection. The presence of prion disease-specific, abnormal accumulations of PrP (referred to as PrP^d^), which occur only in the tissues of affected animals, was detected by IHC (4, 5, 11, 13, 16, 19, 41–43). However, since the IHC analysis cannot unequivocally discriminate between PrP^Sc^ and cellular PrP^C^, paraffin-embedded tissue immunoblot analysis of adjacent membrane-bound sections was also used to confirm that these PrP^d^ aggregates contained prion disease-specific, relatively proteinase K (PK)-resistant PrP^Sc^. As anticipated, abundant PrP^Sc^ accumulations were detected in association with FDC (CD21/35^+^ cells) in Peyer's patches of control *Prnp*^ΔIEC^ mice (Fig. 3a, arrows). Abundant FDC-associated PrP^Sc^ accumulations were also detected in Peyer's patches of βNF-treated *Prnp*^ΔIEC^ mice.

Consistent with the IHC data (Fig. 3a), high levels of prion infectivity were detected in Peyer's patches of mice from each control group (median infectivity level, 6.0 to 6.6 log_10_ intracerebral [i.c.] 50% infectious dose [ID_50_] units/g; *n* = 2 to 4 mice/group) (Fig. 3b). IEC-restricted *Prnp* ablation did not influence the early accumulation of infectious prions within Peyer's patches, as high levels of prion infectivity were also detected in tissues from βNF-treated *Prnp*^ΔIEC^ mice (median infectivity level, 6.1 log_10_ i.c. ID_50_ units/g; *n* = 4 mice) (Fig. 3b).

Within weeks after oral exposure, high levels of ME7 scrapie prions first accumulate on FDC in Peyer's patches and subsequently are disseminated via the blood and lymph to most other lymphoid tissues, including the MLN and spleen (4, 5, 11, 13, 16, 18, 19, 44). The levels of prion infectivity detected in the MLN and spleens from mice from each treatment and control group were also similar (Fig. 3c and d, respectively).

These data clearly show that IEC-restricted *Prnp* ablation does not affect the early accumulation of orally acquired prions within Peyer's patches or their subsequent dissemination to the MLN or spleen.

### IEC-restricted *Prnp* ablation does not influence oral prion disease susceptibility.

Female *Prnp*^ΔIEC^ mice were treated with βNF for 5 days to ablate *Prnp* expression in IEC, and 14 days later, they were subsequently orally exposed to ME7 scrapie prions. Untreated *Prnp*^ΔIEC^ mice, vehicle-treated *Prnp*^ΔIEC^ mice, and βNF-treated *Prnp*^F/F^ (Cre-deficient) mice were used as controls. As anticipated, all of the orally exposed untreated *Prnp*^ΔIEC^ (control) mice succumbed to clinical prion disease (mean survival time of 307 ± 23 days; median of 300 days; *n* = 10/10) (Table 1). Furthermore, IEC-restricted *Prnp* ablation did not affect disease duration (survival times) or susceptibility, as all of the βNF-treated *Prnp*^ΔIEC^ mice also succumbed to clinical prion disease with similar survival times (mean of 306 ± 11 days; median of 306 days; *n* = 12/12; *P* = 0.673 by one-way analysis of variance [ANOVA] with Dunnett's posttest) (Table 1).

**TABLE 1:** *Prnp* deficiency in the gut epithelium does not influence oral prion disease susceptibility

Mouse model^*a*^	Mean survival time (days) ± SD^*b*^	Median survival time (days)	No. of animals with clinical disease/total no. of animals tested	No. of animals with histopathological signs of prion disease in brain (spongiform encephalopathy)/total no. of animals tested
*Prnp*^ΔIEC^	307 ± 23	300	10/10	10/10
*Prnp*^ΔIEC^ + Veh	303 ± 12	303	10/10	10/10
*Prnp*^ΔIEC^ + βNF	308 ± 11	306	12/12	12/12
*Prnp*^F/F^ + βNF	313 ± 19	305	9/9	9/9

a Where indicated, mice were given daily intraperitonal injections of β-napthoflavone (βNF) or corn oil (vehicle control [Veh]) for 5 days. Mice were orally exposed to ME7 scrapie prions 14 days after the last treatment.

b Duration from the time of injection with prions to culling at the clinical endpoint. No statistical differences in survival times were observed between groups (*P* = 0.673 by one-way ANOVA with Dunnett's posttest).

**FIG 4:**
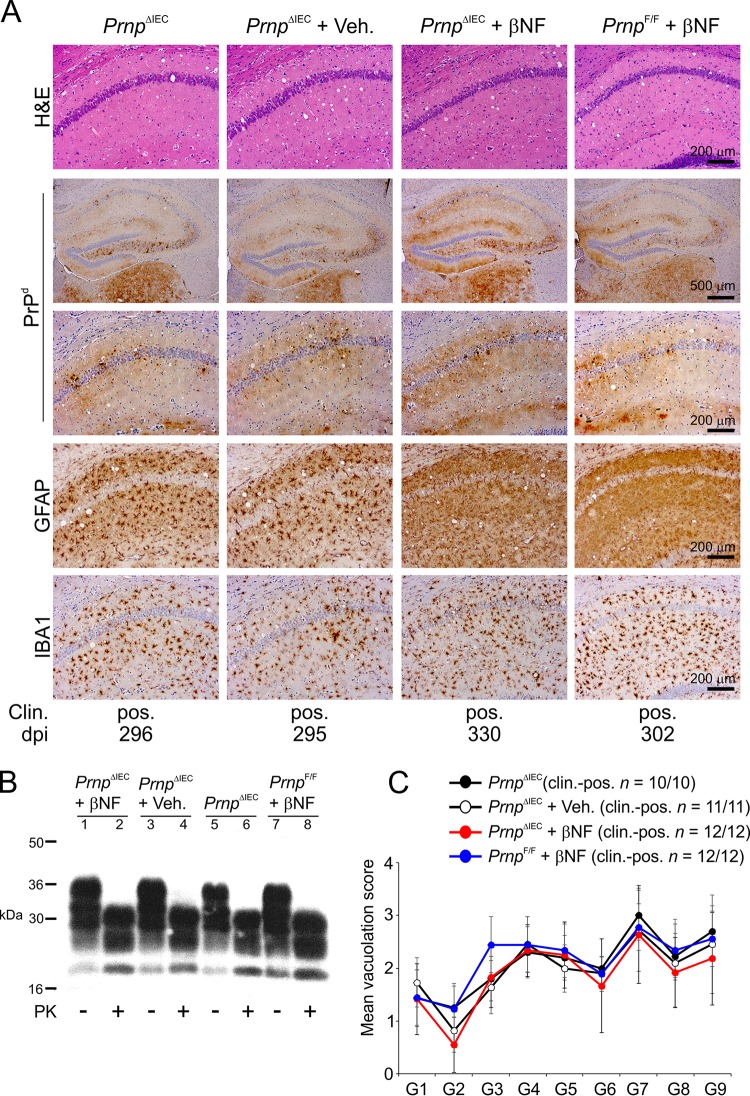
Intestinal epithelial cell-restricted *Prnp* ablation does not influence development of the histopathological signs of prion disease in the brains of clinically affected mice. Female *Prnp*^ΔIEC^ mice were treated with βNF for 5 days to specifically ablate *Prnp* expression in intestinal epithelial cells. Untreated *Prnp*^ΔIEC^ mice and *Prnp*^ΔIEC^ mice treated with the vehicle alone (Veh.) were used as controls. Fourteen days later, the mice were orally exposed to ME7 scrapie prions and culled when they succumbed to clinical prion disease. (A) High levels of spongiform pathology (hematoxylin and eosin [H&E] stain), heavy accumulations of disease-specific PrP (PrP^d^) (brown), reactive astrocytes expressing GFAP (brown), and active microglia expressing Iba-1 (brown) were detected in the brains of all orally exposed mice with clinical prion disease. Clin., clinical prion disease status; pos., clinically positive. Individual survival times are shown (dpi, days postinfection). Sections were counterstained with hematoxylin to detect cell nuclei (blue). (B) Immunoblot analysis of brain tissue homogenates confirms the presence of high levels of prion-specific, relatively proteinase K (PK)-resistant PrP^Sc^ within the brains of the clinically affected mice from each group. Samples were treated in the presence (+) or absence (−) of PK before electrophoresis. After PK treatment, a typical three-band pattern was observed between molecular mass values of 20 and 30 kDa, representing unglycosylated, monoglycosylated, and diglycosylated isomers of PrP (in order of increasing molecular mass). (C) The severity and distribution of the spongiform pathology (vacuolation) within each brain were scored on a scale of 1 to 5 in nine gray matter areas: dorsal medulla (G1), cerebellar cortex (G2), superior colliculus (G3), hypothalamus (G4), thalamus (G5), hippocampus (G6), septum (G7), retrosplenial and adjacent motor cortex (G8), and cingulate and adjacent motor cortex (G9). Each point represents the mean vacuolation score ± SD (*n* = 10 to 12 mice/group).

All the brains from the clinically affected mice in each group displayed the characteristic spongiform pathology (vacuolation), PrP^Sc^ accumulation, astrogliosis, and microgliosis, which is associated with terminal infection with ME7 scrapie prions (Fig. 4A and B). The severity and distribution of the spongiform pathology were also similar in the brains of the clinically affected mice from each group (Fig. 4C).

Together, these data clearly show that efficient prion neuroinvasion after oral exposure occurs independently of PrP^C^ expression in IEC in the small intestine.

## DISCUSSION

The initial transport of prions across the gut epithelium by M cells into small intestinal Peyer's patches is essential to establish efficient infection after oral exposure (13, 16). However, whether the uptake and translocation of prions across the gut epithelium involves a specific receptor is uncertain. Treatments that prevent the initial replication of prions within the GALT impede the spread of prions to the brain and reduce disease susceptibility (4, 13, 16, 18). Thus, the identification of the molecular factors that facilitate the uptake of prions into the GALT will help the design of novel intervention strategies and enhance our understanding of the factors that influence the risk of infection. Small intestinal M cells express cellular PrP^C^ on their apical surfaces, and this may be used by certain gastrointestinal pathogens as an uptake receptor to infect Peyer's patches (26, 34). Independent IHC-based tracing studies have suggested that orally administered prion protein can be transported across the gut epithelium of PrP^C^-deficient mice (14, 17), but whether the expression of PrP^C^ on IEC populations contributed to the establishment of host infection had not been assessed. Data in the current study clearly show that prion neuroinvasion after oral exposure occurs independently of PrP^C^ expression in small intestinal IEC.

Orally acquired prions first replicate in the small intestinal GALT and subsequently spread to most other secondary lymphoid tissues, including the large intestinal GALT. Since oral prion disease susceptibility is substantially reduced in the specific absence of the small intestinal GALT (11), this suggests that the early replication of prions within Peyer's patches is essential to establish efficient host infection after oral exposure. The small intestinal GALT also appear to be the important early sites of prion replication in natural host species (45–47). Although we observed highly efficient Cre-mediated gene recombination in intestinal crypts and IEC throughout the small intestines of βNF-treated *Prnp*^ΔIEC^ mice, the efficiency in the colon was lower and presented a mosaic pattern (Fig. 2c) (35). The less efficient *Prnp* ablation in the large intestine was unlikely to have influenced oral prion disease pathogenesis in the current study, as the large intestinal GALT, such as the colonic patches, are not important early sites of prion replication and neuroinvasion (11).

Despite the potentially widespread exposure of the United Kingdom population to BSE-contaminated food in the 1980s, there have fortunately been many fewer clinical cases of variant Creutzfeldt-Jakob disease in humans than the original estimates suggested (48) (178 definite or probable cases, as of 4 May 2018 [http://www.cjd.ed.ac.uk/]). This implies that additional factors could potentially influence an individual's susceptibility to oral prion infection by enhancing or impeding the initial uptake of prions from the gut lumen. In support of this hypothesis, we have shown that stimuli that increase the density of M cells in the gut epithelium also increase oral prion disease susceptibility approximately 10-fold by enhancing the uptake of prions into Peyer's patches (16). The expression level of PrP^C^ in host cells such as neurons and FDC directly influences survival times of prion-infected mice (43, 49–51). Acute mucosal inflammation following oral infection with *S*. Typhimurium and treatment with dextran sodium sulfate have each been shown to enhance PrP^C^ expression in the large intestine, implying the potential to enhance oral prion disease pathogenesis and susceptibility (52, 53). Conversely, PrP^C^ expression was reported to be downregulated in the small intestines of mice treated with the nonsteroidal anti-inflammatory drug indomethacin and coincided with a modest increase in survival time after oral exposure to ME7 scrapie prions (54). Although the cellular sites of PrP^C^ expression were not determined in the above-mentioned studies, our data suggest that the magnitude of PrP^C^ expression in IEC throughout the small intestine is unlikely to be an important factor which influences the risk of oral prion disease susceptibility.

In sheep with natural scrapie (55) or orally exposed to BSE prions (56), prion accumulation is first detected in the palatine tonsils in addition to Peyer's patches. Natural prion disease-susceptible host species such as sheep and cervids also have highly developed olfactory systems, which they use to detect food, select mates, and sense predators. A series of experimental studies in rodents and sheep showed that prion infections can be established via the nasal cavity (57–59). Thus, it cannot be excluded that soil-bound prions might also be inhaled and infect the host as the animal forages for food. Although M cells are present in the epithelia covering the nasal-associated lymphoid tissue (60), studies in hamsters indicate that this prion uptake across the nasal epithelium occurs independently of M cells (61). Whether prion uptake across the mucosal surfaces in the upper gastrointestinal and upper respiratory tracts of natural host species is also PrP^C^ independent remains to be determined.

In conclusion, we show that oral prion disease neuroinvasion occurs independently of PrP^C^ expression in IEC in the small intestine. Whether prions exploit other receptors on the apical surfaces of M cells to establish host infection is uncertain. The specific targeting of vaccine antigens to M cells has been shown to be an effective method to induce protective antigen-specific mucosal immunity (62). Mucosal immunization has also been shown to provide promising protection against oral prion infections in mice (63) and white-tailed deer (64). Thus, a thorough understanding of the mechanisms that prions exploit to establish infection within the GALT may help to identify important factors which influence disease susceptibility or identify novel targets for prophylactic intervention.

## MATERIALS AND METHODS

### Mice.

The following mouse strains were used in this study where indicated: *Cyp1a1*-Cre (35); the ROSA26^F/F^ reporter strain (40); and *Prnp*^F/F^ mice (strain *Prnp*^tm2Tuzi^), which have *LoxP* sites flanking exon 3 of the *Prnp* gene (39). C57BL/Dk mice were also used where indicated. All mice were bred and maintained under specific-pathogen-free (SPF) conditions. All studies and regulatory licenses were approved by the institute's ethics committee and carried out under the authority of a United Kingdom Home Office project license. Prior to the use of mice in experiments, the genotype of each mouse was confirmed by PCR analysis of tail DNA (Table 2).

**TABLE 2:** PCR primers used to confirm mouse genotypes^*a*^

Allele	Description	Primer sequence	Product size (bp)
*Cre*	Fwd	CGAGTGATGAGGTTCGCAAGAACC	786
	Rev	GCTAAGTGCCTTCTCTACACCTGC	
*LacZ*	Fwd	TACCACAGCGGATGGTTCGG	300
	Rev	GTGGTGGTTATGCCGATCGC	Recombined *Prnp^F^*, 344
*Prnp^flox^*	1	AATGGTTAAACTTTCGTTAAGGAT	*Prnp^F^*, 210
	2	GCCGACATCAGTCCACATAG	*Prnp^+^*, 167
	3	GGTTGACGCCATGACTTTC	
*Prnp^+^*	Fwd	TCATCCCACGATCAGGAAGATGAG	600
	Rev	ATGGCGAACCTTGGCTACTGGCTG	

a Fwd, forward primer; Rev, reverse primer; Recombined *Prnp^F^*, Cre-mediated DNA recombined allele.

### β-Naphthoflavone treatment.

Where indicated, mice were given five daily intraperitoneal injections of β-naphthoflavone (80 mg/kg of body weight; Sigma-Aldrich, Poole, UK) dissolved in corn oil (Sigma-Aldrich) and analyzed 14 days after the last injection or used in subsequent experiments. Where indicated, some mice received either corn oil alone (vehicle) or no treatment as controls.

### Histological assessment of *LacZ* expression.

Tissues were first immersed in *LacZ* fixative (phosphate-buffered saline [PBS] [pH 7.4] containing 2% paraformaldehyde, 0.2% glutaraldehyde, 0.02% Nonidet P-40, 0.01% sodium deoxycholate, 5 mM EGTA, and 2 mM MgCl_2_) and washed in *LacZ* wash buffer (PBS [pH 7.4] containing 0.02% Nonidet P-40, 0.01% sodium deoxycholate, and 2 mM MgCl_2_). Tissues were subsequently incubated in 15% (wt/vol) sucrose in PBS overnight, followed by a further overnight incubation in 30% (wt/vol) sucrose in PBS, and embedded in Tissue-Tek OCT compound (Bayer PLC, Newbury, UK). Serial sections (thickness, 8 mm) were cut on a cryostat and stained overnight with *LacZ* staining solution (Glycosynth, Warrington, UK). The staining reaction was stopped by washing in *LacZ* wash buffer followed by distilled water. Sections were counterstained with nuclear fast red (Vector Laboratories, Peterborough, UK). Intestinal whole mounts were prepared luminal side up, as described previously (65), and fixed in ice-cold 2% formaldehyde–0.2% glutaraldehyde in PBS (pH 7.4) for 1 h before overnight incubation in *LacZ* staining solution.

### Prion exposure and disease monitoring.

For oral exposure, mice were fed individual food pellets dosed with 50 μl of a 1.0% (wt/vol) dilution of scrapie brain homogenate (containing approximately 4.6 log_10_ i.c. ID_50_ units) prepared from mice terminally affected with ME7 scrapie prions according to our standard protocol (11, 16, 19). During the dosing period, mice were individually housed in bedding- and food-free cages, with water provided ad libitum. A single prion-dosed food pellet was then placed in the cage. The mice were returned to their original cages (with bedding and food ad libitum) as soon as the food pellet was observed to have been completely ingested. The use of bedding- and additional food-free cages ensured easy monitoring of consumption of the prion-contaminated food pellet. Following prion exposure, mice were coded, assessed weekly for signs of clinical disease, and culled at a standard clinical endpoint. The clinical endpoint of disease was determined by rating the severity of clinical signs of prion disease exhibited by the mice. Mice were clinically scored as “unaffected,” “possibly affected,” and “definitely affected” using standard criteria that typically are present in mice with terminal ME7 scrapie prion disease. Clinical signs following infection with the ME7 scrapie prions may include weight loss; starry coat; hunched, jumpy behavior (at early onset) progressing to limited movement; upright tail; wet genitals; decreased awareness; discharge from eyes/blinking eyes; and ataxia of hind legs. The clinical endpoint of disease was defined in one of the following ways: (i) the day on which a mouse received a second consecutive “definite” rating, (ii) the day on which a mouse received a third “definite” rating within four consecutive weeks, or (iii) the day on which a mouse was culled in extremis. Prion diagnosis was confirmed by histopathological assessment of the magnitude of the spongiform pathology (vacuolation) in nine distinct gray matter regions of the brain as described previously (66).

For bioassays of prion infectivity, individual tissues were prepared as 10% (wt/vol) homogenates, and 20 μl was injected i.c. into each of 4 recipient C57BL/Dk indicator mice. The prion infectivity titer in each sample was determined from the mean incubation period in the indicator mice, by reference to a dose/incubation period-response curve for ME7 scrapie-infected spleen tissue serially titrated in C57BL/Dk indicator mice (67).

### Immunohistochemistry.

For the detection of disease-specific PrP (PrP^d^) in intestines and brains, tissues were fixed in periodate-lysine-paraformaldehyde fixative and embedded in paraffin wax. Sections (thickness, 6 μm) were deparaffinized and pretreated to enhance the detection of PrP^d^ by hydrated autoclaving (15 min, 121°C, hydration) and subsequent immersion in formic acid (98%) for 5 min. Sections were then immunostained with 1B3 PrP-specific polyclonal antibody (pAb). For the detection of FDC in intestines, deparaffinized sections were first pretreated with target retrieval solution (Dako) and subsequently immunostained with anti-CD21/35 (clone 7G6; BD Biosciences). Paraffin-embedded tissue immunoblot analysis was used to confirm that the PrP^d^ detected by immunohistochemistry was proteinase K-resistant PrP^Sc^ (68). Membranes were subsequently immunostained with 1B3 PrP-specific pAb.

For the detection of astrocytes, brain sections were immunostained with anti-glial fibrillary acidic protein (GFAP; Dako, Ely, UK), and to detect microglia sections, they were immunostained with anti-ionized calcium-binding adaptor molecule 1 (Iba-1; Wako Chemicals GmbH, Neuss, Germany).

Following the addition of primary antibodies, biotin-conjugated species-specific secondary antibodies (Stratech, Soham, UK) were applied, and immunolabeling was revealed using either alkaline phosphatase conjugated to the avidin-biotin complex (Vector Laboratories, Peterborough, UK), visualized using Vector red, or horseradish peroxidase (HRP) conjugated to the avidin-biotin complex (Vector Laboratories), visualized with 3,3'-diaminobenzidine (Sigma). Sections were counterstained with hematoxylin to distinguish cell nuclei.

### Immunoblot detection of PrP^Sc^.

Brain homogenates (10%, wt/vol) were prepared in NP-40 lysis buffer (1% NP-40, 0.5% sodium deoxycholate, 150 mM NaCl, 50 mM Tris HCl [pH 7.5]) and incubated at 37°C for 1 h with 20 μg/ml PK. Digestions were halted by the addition of 1 mM phenylmethylsulfonyl fluoride. Samples were then subjected to electrophoresis through 12% Tris-glycine polyacrylamide gels (NuPAGE; Life Technologies) and transferred to polyvinylidene difluoride (PVDF) membranes by semidry blotting. PrP was detected using anti-mouse PrP-specific monoclonal antibody (mAb) 7A12 (69), followed by horseradish peroxidase-conjugated goat anti-mouse antibody (Jackson ImmunoResearch), and visualized by chemiluminescence (BM chemiluminescent substrate kit; Roche, Burgess Hill, UK).

### Statistical analyses.

Unless indicated otherwise, data are presented as means ± standard deviations (SD), and significant differences between groups were sought by Student's *t* test. *P* values of <0.05 were accepted as significant.
